# Supplementary material for: Low-dimensional heat conduction in surface phonon polariton waveguide
Source: Nat Commun. 2023 Dec 12;14:8242. doi: 10.1038/s41467-023-43736-8 (PMC10716141; doi:10.1038/s41467-023-43736-8)
Supplement: Supplementary file 1 — Supplementary Information [file 41467_2023_43736_MOESM1_ESM.pdf]

Supplementary Information

Low-dimensional Heat Conduction in Surface Phonon Polariton Waveguide

Pei et al.

Supplementary Note 1: Device fabrication process

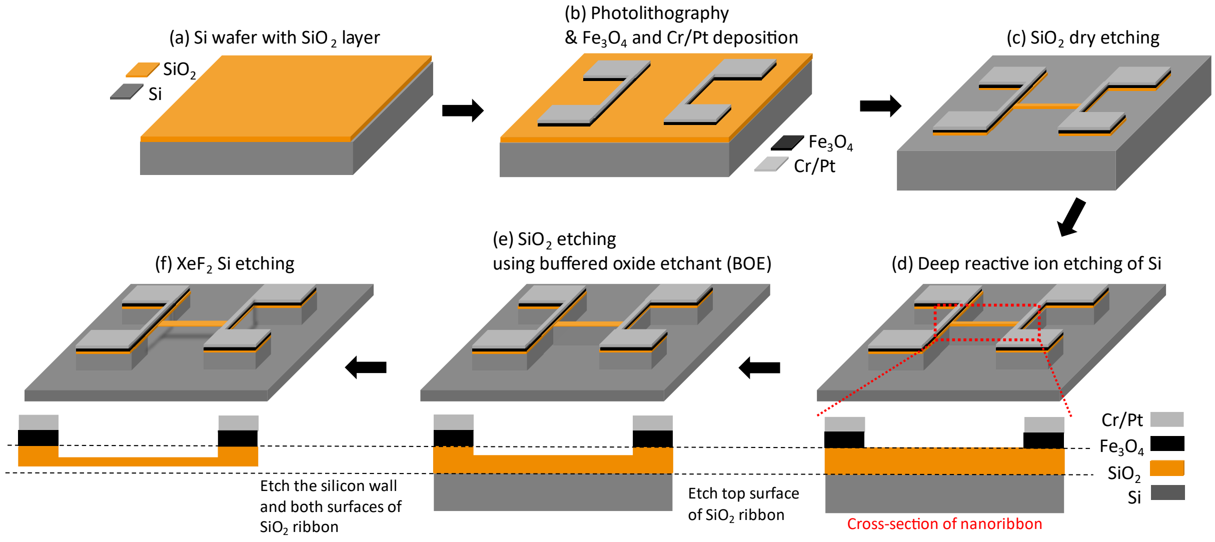

Fig. S1. Device fabrication process. a, The fabrication of NRs starts with a silicon wafer having a 100 nm wet thermal oxide layer on top. **b,** A layer of photoresist (NR9-3000PY) is spin-coated onto the wafer. Then, the photolithography (Heidelberg MLA150) is used to pattern the Fe_3O_4 and electrodes layer. After hard baking and photoresist development, the wafer is deposited with Fe_3O_4 (350 nm), Cr (5 nm), and Pt (110 nm) in sequence using sputtering (Denton Discovery 18 Sputter System). At last, the wafer is soaked in Resist Remover RR4 to do lift-off. **c,** Photoresist is used as an etching mask to define the SiO_2 NRs and cover the electrodes. The SiO_2 dry etching process (Oxford Plasmalab P80, CHF_3 and Ar gas) etches other parts of the SiO_2 on the Si wafer. **d,** Another lithography step provides an etching mask with a slightly larger area than the NR and electrodes, followed by Si deep reactive ion etching (Oxford Plasmalab P100 RIE/ICP, SF_6 gas), which creates a step between SiO_2 NRs and Si substrate. Resist Remover RR4 and O_2 plasma are used to remove photoresist and clean the photoresist residues. **e,** The sample is placed in buffered oxide etchant (BOE) to get thinner SiO_2 NRs. This process only etches the top surface of SiO_2 NRs. The thickness of the SiO_2 NRs can be controlled by adjusting the etching time. **f,** The sample is etched by XeF_2 (Xactix XeF_2 Etcher) to release the device and make suspended structures. XeF_2 possesses high selectivity between Si and SiO_2 (> 1000:1). XeF_2 etches the Si wall under the NRs, resulting in suspended SiO_2 NRs. During this process, however, XeF_2 also etches SiO_2 NR slightly. The exact thickness of the suspended SiO_2 NRs is determined from frequency-dependent thermal transport measurement on the NRs, which yields heat capacity (and cross-section area) of the NR (see Supplementary Note 6).

Supplementary Note 2: SEM of the measured NR samples

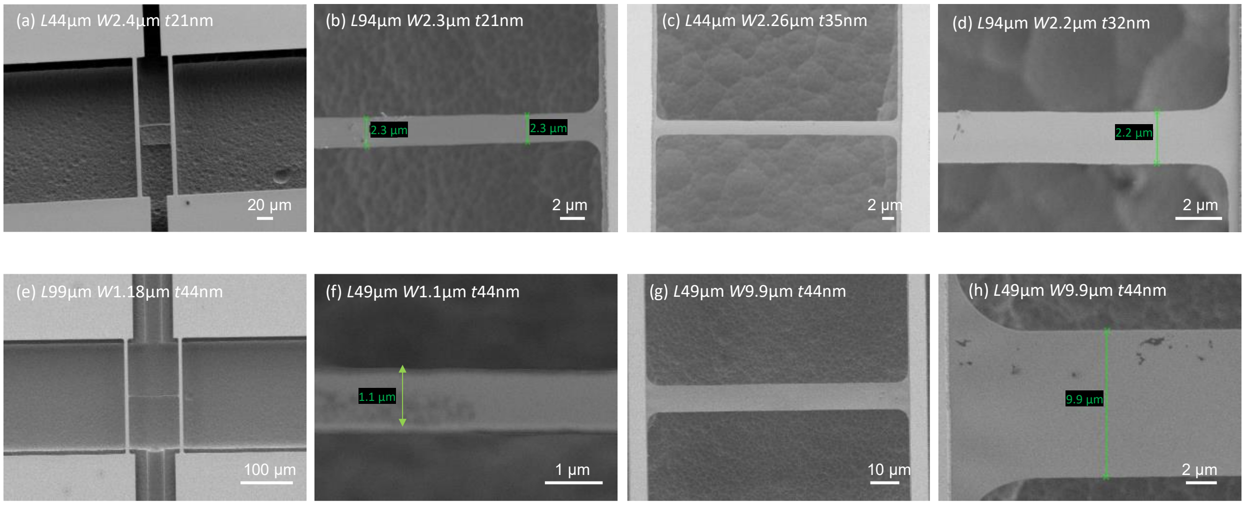

Fig. S2. SEM images of SiO_2 NR with different widths and lengths. a, L = 44 μm, W = 2.4 μm, t = 21 nm. **b,** L = 94 μm, W = 2.3 μm, t = 21 nm. **c,** L = 44 μm, W = 2.26 μm, t = 35 nm. **d,** L = 94 μm, W = 2.2 μm, t = 32 nm. **e,** L = 99 μm, W = 1.18μm, t = 44nm. **f,** L = 49 μm, W = 1.1 μm, t = 44 nm. **g,** L = 49 μm, W = 9.9 μm, t = 44 nm. **h,** L = 49 μm, W = 9.9 μm, t = 44 nm.

Supplementary Note 3: NR thickness measurement

The original SiO_2 layer thickness is 100 nm from the thermal oxide layer of the Si wafers. The thickness of the NRs after the buffered oxide etchant (BOE, containing buffered HF) etching process can be measured using a surface profiler (DektakXT). We denote this thickness as t_0. Note that this thickness is further reduced slightly to the final thickness (t) of the suspended NRs due to the XeF_2 etching, which has a very low but non-negligible etching rate on SiO_2. As shown in Fig. S3c, the NR thickness after a 30s BOE etching process for the sample of L = 49 μm, W = 9.9 μm, t = 44 nm is around 62 nm, i.e., t_0= 62 nm. The chip has a Pt marker on top of SiO_2 as shown in Fig. S3d, which was used to measure the reduction of the top surface SiO_2 thickness (δ_t) during the XeF_2 etching process by measuring the thickness difference before and after the XeF_2 etching. For the sample of L = 49 μm, W = 9.9 μm, t = 44 nm, we found δ_t = 15 nm. For the SiO_2 NRs during the fabrication steps of XeF_2 etching (from Fig.S1e to Fig. S1f), initially only the top surface of SiO_2 NR was etched by XeF_2 and then both of the top and bottom surfaces were etched by XeF_2 after the NR has been suspended. Therefore, the suspended NR thickness will be between $t_0 - \delta_t$ and $t_0 - 2\delta_t$ depending on sample size and etching cycles. For the sample with L = 49 μm, W = 9.9 μm, t = 44 nm, the final NR thickness (t) is expected to be between 32 – 47 nm (i.e., between (62 – 30) nm and (62 – 15) nm). The actual thickness ($t = 44$ nm) obtained from the frequency-dependent measurement (Supplementary Note 6) is within this expected range.

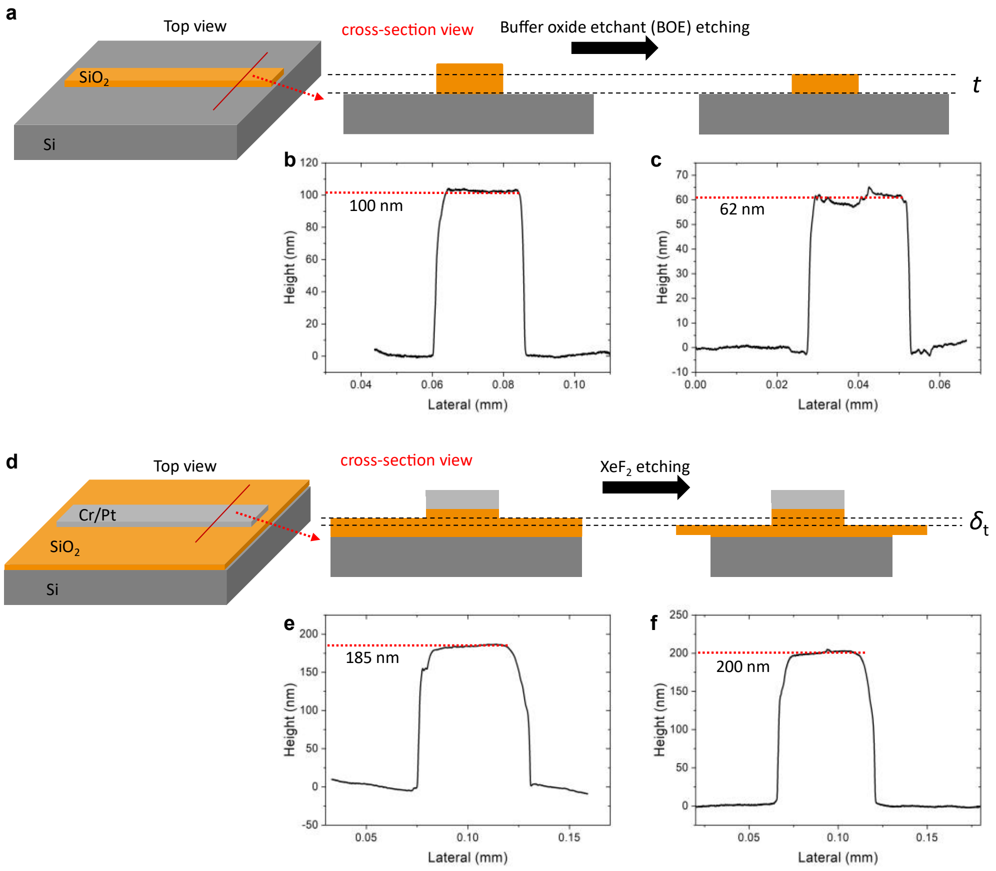

Fig. S3. NR thickness measurement. a, Schematic of SiO_2 NR thickness reducing after BOE etching, the red line is the surface profiler scan route. We denote the thickness after the BOE etching as t_0. **b c,** Surface profile measurement results for the sample of L = 49 μm, W = 9.9 μm, t = 44 nm before and after BOE etching. The thickness is reduced from the original thermal oxide layer thickness of 100 nm to 62 nm. **d,** Schematic of Pt marker on SiO_2. **e f,** Surface profile measurement results for the sample before and after XeF_2 etching, showing the 15 nm etching (δ_t) of the SiO_2 layer by XeF_2. This is a very small but non-negligible etching of SiO2 considering that the Si is etched by more than 10 μm. For the SiO_2 NR, the thickness reduction due to the XeF_2 etching is expected to be between δ_t and $2\delta_t$ because the NR is suspended during the etching process, after which the bottom surface of the NR is also etched.

Supplementary Note 4: Thermal conductivity measurement platform

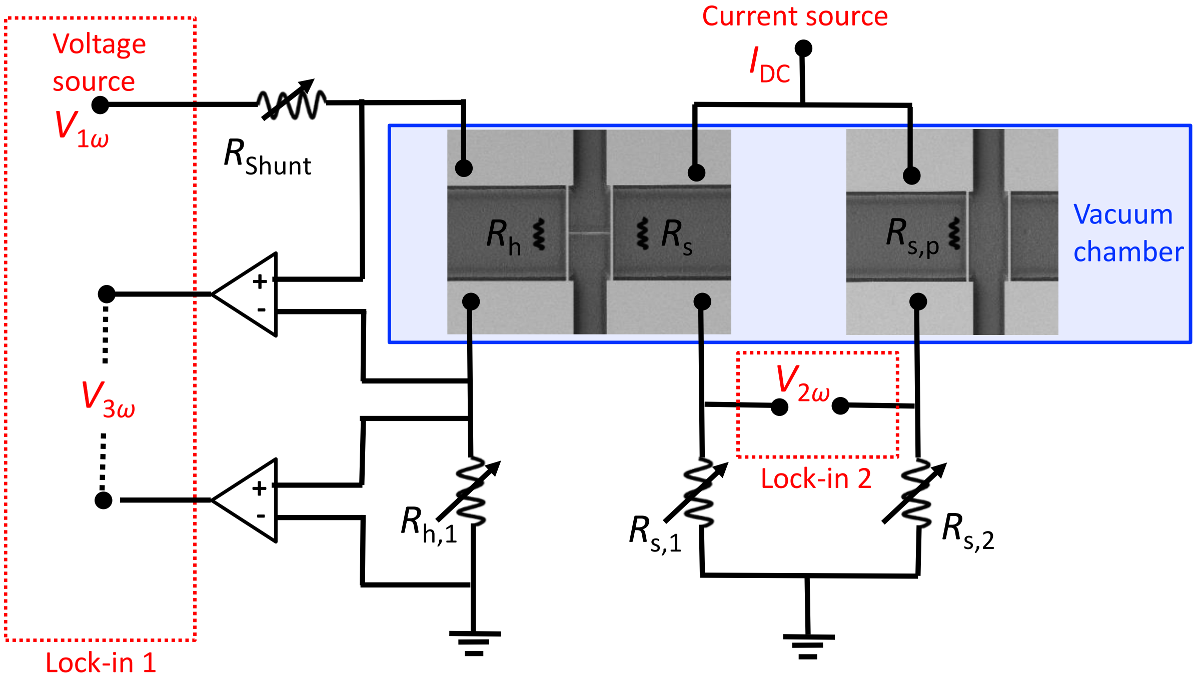

Fig. S4. Schematic of the thermal conductivity measurement platform

The SiO_2 NR thermal conductivity measurement platform (as shown in Fig. S4) is based on the AC heating and lock-in thermometry scheme. Its thermal conductance resolution can reach the order of 10s pw/K[1,2]. An AC voltage ($V_{1\omega}$) at a modulated frequency ω is applied on one of the SiO_2/Pt beams (denoted as the heating beam, R_h). The frequency is set at 0.4847 Hz so that the heat penetration depth is much longer than the NR length. The joule heating created a second harmonic temperature rise in the heating beam and consequently a third harmonic voltage signal ($V_{h,3\omega}$) due to the Pt beam resistance change (($\Delta R_{h,2\omega}$). A lock-in amplifier (Stanford Research SR830) collects the $V_{3\omega}$ signal which can be used to calculate the temperature rise (ΔT_h) in the center of the heating beam (Eq. 1). Party of the heat on the heating beam, denoted as Q_s, will transfer to the other beam (sensing beam, R_s) through the SiO_2 NR which yields a second harmonic temperature rise in the sensing beam (ΔT_s). A beam of another sample on the same chip is served as the pair resistor ($R_{s,p}$) and forms a Wheatstone bridge scheme together with R_s, R_1, and R_2. This on-chip Wheatstone bridge circuit could effectively reject common mode noise and thus improve the measurement sensitivity [2]. A small direct current (I_{DC}) is applied to the bridge circuit and another lock-in amplifier is used to measure the 2nd harmonic bridge voltage ($V_{s,2\omega}$). Then the temperature rise in the middle of the sensing beam (ΔT_s) can be calculated using Eq. S2[1].

$$\Delta T_{\mathrm{h}} = 3\,\frac{V_{\mathrm{h},3\omega}}{I_{\omega}}\left(\frac{dR_h}{dT}\right)^{-1} \tag{S1}$$

$$\Delta T_{\mathrm{s}} = \sqrt{2}\,\frac{V_{\mathrm{s},2\omega}(R_{\mathrm{s}}+R_{\mathrm{s,p}}+R_1+R_2)}{I_{\mathrm{DC}}R_2}\left(\frac{dR_{\mathrm{s}}}{dT}\right)^{-1} \tag{S2}$$

where I_{ω} is the current flowing in the heating beam, $\frac{dR_{\mathrm{h}}}{dT}$ and $\frac{dR_{\mathrm{s}}}{dT}$ are the temperature coefficient of resistance (TCR) of the heating and sensing beams, respectively.

The heat flux from the heating to the sensing beam (Q_{s}) is the same as heat transfer from the middle of the sensing beam to the substrate. Thus, the apparent thermal conductance of the SiO_2 NR (G_{a}) can be determined by:

$$G_{\mathrm{a}}(\Delta T_{\mathrm{h}} - \Delta T_{\mathrm{s}}) = Q_{\mathrm{s}} = G_{\mathrm{b}}\Delta T_{\mathrm{s}} \tag{S3}$$

$$G_{\mathrm{a}} = \frac{G_{\mathrm{b}}\Delta T_{\mathrm{s}}}{(\Delta T_{\mathrm{h}}-\Delta T_{\mathrm{s}})} \cong G_{\mathrm{b}}\,\frac{\Delta T_{\mathrm{s}}}{\Delta T_{\mathrm{h}}} \tag{S4}$$

where G_{b} is the thermal conductance of sensing beam. ΔT_{s} is several orders of magnitude smaller than ΔT_{h}, so ΔT_{h}- ΔT_{s} can be approximated as ΔT_{h} in Eq. S4.

Supplementary Note 5: Principle of the thermal conductivity measurement

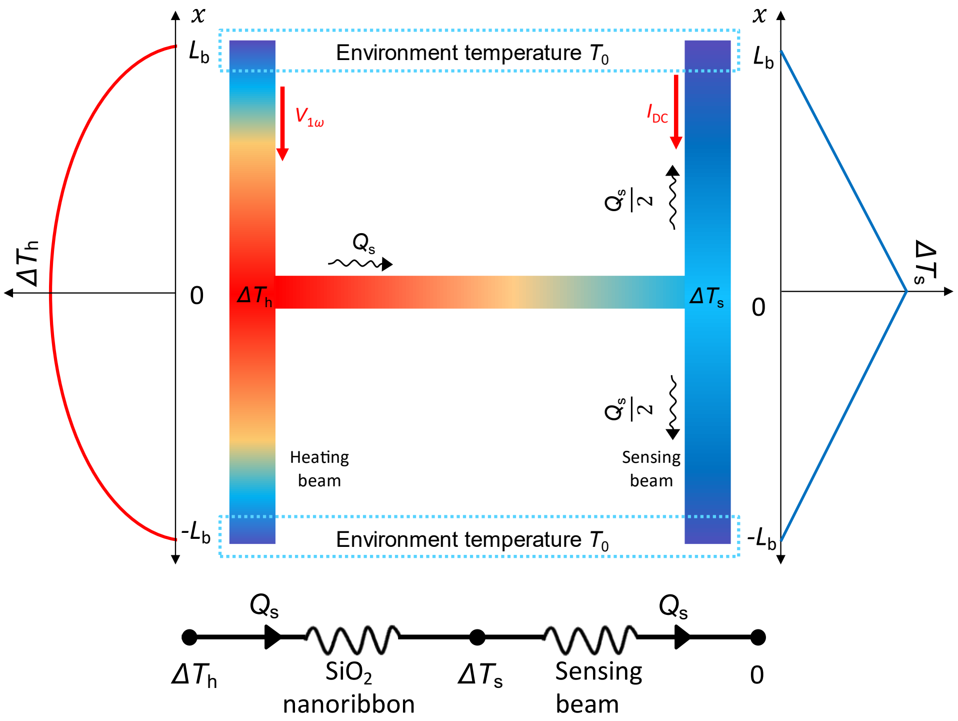

Fig. S5. Temperature distribution on the measurement device, including the left heating and right sensing beams and the NR bridging the two.

Fig. S5 shows the temperature distribution on the sample. For both the heating and the sensing beams, the ends of the beams have the same temperature as the Si substrate. The temperature rise distribution along the heating beam due to the Joule heating can be written as follows [1]

$$\Delta T_{\rm h}(x) = -\frac{q_{\rm h}}{2k_{\rm b}}x^2 + \frac{q_{\rm h}}{2k_{\rm b}}L_{\rm b}^2 \quad \text{(S5)}$$

where $q_{\rm h}$ is the internal heat source per unit volume due to the Joule heating, $q_{\rm h} = \frac{I_{\rm h}^2 R_{\rm h}}{2L_{\rm b}S_{\rm b}}$, $I_{\rm h}$ is the root-mean-squared (RMS) value of the current on the heating beam, $R_{\rm h}$ is the heating beam resistance, $L_{\rm b}$ is the half beam length, $S_{\rm b}$ is the beam cross-section area, $k_{\rm b}$ is the thermal conductivity of the beam. The highest temperature is in the middle of the heating beam: $\Delta T_{\rm h}|_{x=0} = \frac{q_{\rm h}}{2k_{\rm b}}L_{\rm b}^2$. As for the sensing beam, the temperature distribution is linear since there is only heat conduction from the middle of the beam to the end.

In the thermal conductivity measurements, we applied different AC voltages on the heating beam, which give different heating power $Q_{\rm h} = I_{\rm h}^2 R_{\rm h}$. Fig. S6 shows the measured $\Delta T_{\rm h}$ and $\Delta T_{\rm s}$ with different $Q_{\rm h}$ at 300 K and 550 K. We use linear fitting to get slopes of $\Delta T_{\rm h}/Q_{\rm h}$ and $\Delta T_{\rm s}/Q_{\rm h}$ and use the ratio of the slope to yield $\frac{\Delta T_{\rm s}}{\Delta T_{\rm h}}$, which is used to determine the thermal conductance of the NRs (as shown in Eq. S4).

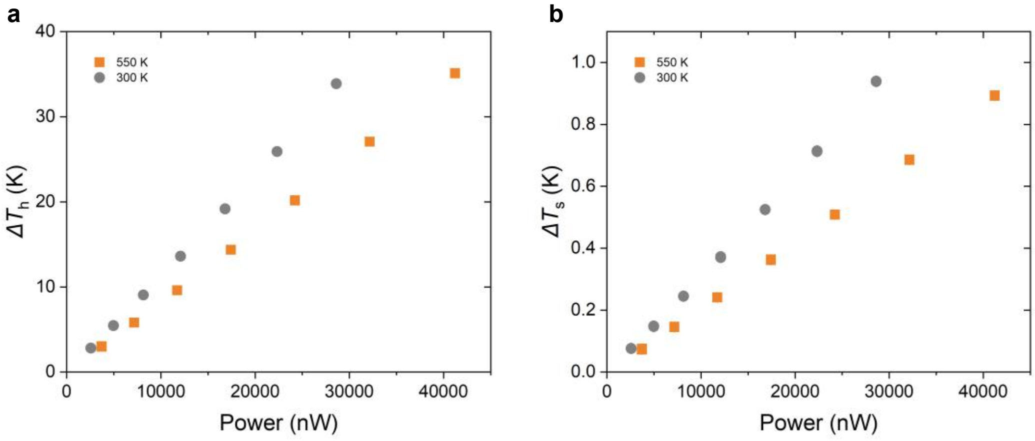

Fig. S6. Heating and sensing beam temperature rise (ΔT_{h}, ΔT_s) versus input power. **a,** ΔT_{h} and **b,** ΔT_{s} with different Q_{h} at 300 K and 550 K for the NR sample with L = 49 μm, W = 9.9 μm, and t = 44 nm.

Supplementary Note 6: Frequency dependent measurements for NR thickness fitting

In order to accurately determine the thickness of SiO_2 NR, we also did frequency dependent measurements for our samples. By modulating the AC heating current frequency, the thermal penetration depth (L_p) will also be changed. In the low-frequency regime, L_p is longer than the NR length (L), which is the frequency range we used in our NR thermal conductivity measurement. As frequency increases, the L_p will be decreasing. This is the frequency regime we can use to determine the heat capacity (or thermal diffusivity) of the NR sample.

For both the NR and the sensing beams, we can use the following equation to analyze the heat transfer:

$$\frac{\partial \Delta T(x,t)}{\partial t} = \alpha \frac{\partial^2 \Delta T(x)}{\partial x^2} \tag{S6}$$

where α is the thermal diffusivity. At angular frequency ω, by setting $\Delta T(x,t) = \Delta T(x) e^{j\omega t}$, Eq. S6 can be written as

$$j\omega \Delta T(x) e^{j\omega t} = \alpha \frac{d^2 \Delta T(x)}{dx^2} e^{j\omega t} \tag{S7}$$

The general solution for Eq. S7 is

$$\Delta T_i(x) = C_i' \cosh(a_i x) + C_i'' \sinh(a_i x) \tag{S8}$$

where $a^2 = \frac{j\omega}{\alpha}$. C_i' and C_i'' are constants and the subscripts i = 1, 2 are for the sensing beam and the NR, respectively. By applying the corresponding boundary conditions for the beam and the NR, the relation between frequency and temperature rise ratio at the two ends of the NR can be written as [2]

$$\frac{\Delta T_s}{\Delta T_h} = \frac{1}{\frac{k_1 A_1 a_1 \sinh(a_2 L_2)}{k_2 A_2 a_2 \tanh(a_1 L_1)} + \cosh(a_2 L_2)} \tag{S9}$$

where k, A, L are the thermal conductivity, cross-section area and length for the NR and the beam respectively.

In this equation, the only unknown parameter is $A_2 = (W_2 t_2)$. As the width of NR (W_2) can be measured, the thickness of NR (t_2) can be obtained by fitting Eq. S9 to the measured data. Fig. S7a shows the thickness fitting result for a SiO_2 NR, where the experimental data is the measured temperature rise ratio between the sensing and heating side at different frequencies and the solid fitting curves are calculation results using Eq. S9 by assuming different NR thicknesses. This result shows that t_2 = 47 nm yields the best fitting.

To confirm this thickness, we did atomic force microscopy (AFM) imaging on the same NR. We transferred the NR to a polydimethylsiloxane (PDMS) substrate by buckling the sample on the substrate and applying pressure. The NR thickness was then measured by using an AFM and the result is shown in Fig. S7b, with an inset image of the NR scanned by AFM. Both thickness fitting and AFM measurement show the NR thickness is around 47 nm. Additionally, the thermal conductivity of SiO_2 NR calculated using this dimension matches well with bulk SiO_2 value at low temperature, where the role of SPhP heat conduction is negligible.

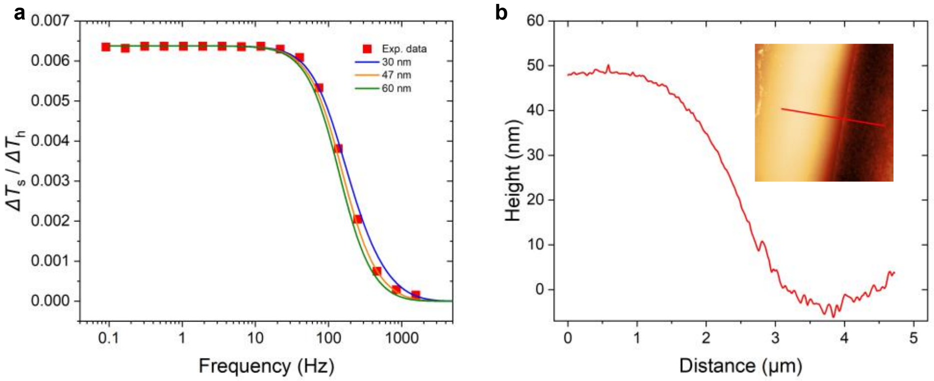

Fig. S7. Combined thermal transport and tomographic measurements to determine NR thickness. a, SiO_2 NR thickness fitting result through frequency-dependent temperature rise measurement. **B,** Atomic Force Microscopy (AFM) thickness measurement result of the same NR; inset is an AFM image of the SiO_2 NR and the red line is the scan route.

Supplementary Note 7: SPhP thermal conductance (G_{SPhP})

We use the Landauer formula to analyze the SPhP thermal conductance (G_{SPhP}). Consider a rectangular SiO_2 NR waveguide with width of *W*, thickness of *t*, length of *L*, and temperature of T_h and T_s at each end, the heat flux Q along this NR is given by the Landauer formula [3]:

$$Q = \sum_{n=1}^{N} \int_{0}^{k_n^{max}} \frac{1}{2\pi} \hbar\omega V[f_\omega(T_h) - f_\omega(T_s)]\tau_n(k)\eta_n(k)dk \tag{S10}$$

where k, ω and $V = \partial\omega/\partial k$, are the wave vector, angular frequency, group velocity of the energy carriers propagating along the nanowire. Consider the energy carriers of SPhP in our case, *k* is the wave vector of SPhP. N is the number of SPhP modes supported by the SiO_2 NR. $f_\omega(T)$ is the Bose-Einstein distribution function, τ_n is the transmission probability of each mode and η_n is the corresponding absorption coefficient.

Considering that the temperature difference of temperature $\Delta T = T_h - T_c \ll (T_h + T_s)/2$, the term $f_\omega(T_h) - f_\omega(T_s)$ reduces to $f_\omega(T_h) - f_\omega(T_s) = \Delta T \partial f_\omega(T)/\partial T$. Then the SPhP thermal conductance can be expressed from $G = Q/\Delta T$ as:

$$G_{SPhP} = \frac{1}{2\pi} \sum_{n=1}^{N} \int_{\omega_n^{min}}^{\omega_n^{max}} \hbar\omega \frac{\partial f_\omega(T)}{\partial T} \tau_n(\omega)\eta_n(\omega)d\omega \tag{S11}$$

where ω_n^{min} and ω_n^{max} stand for the lowest and highest frequencies of each mode. The value of ω_n^{min} and ω_n^{max} are considered as the lower and upper frequency limit of optical phonon of the materials. For example, 7.6 Trad/s and 258 Trad/s are used for SiO_2 in literatures[4]. In experiments like our system, the heating and sensing beam with Pt and Fe_3O_4 are included, which will contribute to the mode excitations and absorption and thus contribute to thermal conductance. Considering that both Pt and Fe_3O_4 are lossy at a broadband frequency covering the whole range of Bose-Einstein distribution at our interested temperature, we can simply assume $\omega_n^{min} = 0$, $\omega_n^{max} = \infty$. Similar thermal excitation beyond the optical phonon range was already reported.[5] If considering the ideal case of $\omega_n^{min} = 0$, $\omega_n^{max} = \infty$, $\tau_n = 1$, and $\eta_n = 1$, then $G_{SPhP} = N \cdot G_0$, where $G_0 = \pi^2 k_B^2 T/3h$ is known as the quantum of thermal conductance.

The thermal conductivity $k = G \cdot L/A$ is then given by:

$$k_{SPhP} = \frac{L}{2\pi A} \sum_{n=1}^{N} \int_{\omega_n^{min}}^{\omega_n^{max}} \hbar\omega \frac{\partial f_\omega(T)}{\partial T} \tau_n(\omega)\eta_n(\omega)d\omega \tag{S12}$$

where $A = tW$ is the area of the cross-section of the NR.

In order to get the thermal conductivity, we first studied the characteristics of the propagation modes in the SiO_2 NR, including the dispersion relation, propagation length and also the mode profile. Here for our purpose, numerical simulations using the mode analysis module in a finite element method based commercial software (COMSOL Multiphysics) were conducted. The SiO_2 waveguide is modeled as a rectangular shape with an exemplary width of W = 10 μm, thickness t = 50 nm with the surrounding medium as vacuum. Fig. S8 shows the solved wave vector and mode profile of quasi-TM and quasi-TE mode. Both quasi-TM and quasi-TE modes show wave vectors very close to the vacuum wave vector k_0, leading to small confinement and long propagation length. An exception is the quasi-TE mode in the metallic region (gray region in Fig. S8). This is because, for quasi-TE mode, the electric field along the direction parallel to the SiO_2 NR exhibits a symmetric distribution. When the permittivity of SiO_2 is shown as metallic, the electric field is highly confined within the metal, resulting in a short SPhP wavelength, a short decay length into the surrounding medium, and a smaller propagation length, due to enhancement of Ohmic losses. The wave vector is discontinuous within the wavelength range of 7.2 μm to 8.3 μm (green region in Fig. S8), which is due to the fact that the refractive index $(n + ik)$ of SiO_2 is less than 1. As a result, the calculated wave vector is also less than k_0, which is physically invalid. Because if $k < k_0$, the fields outside the SiO_2 NR would be radiative in nature and would imply that the system is continually radiating energy out to infinity without a source.

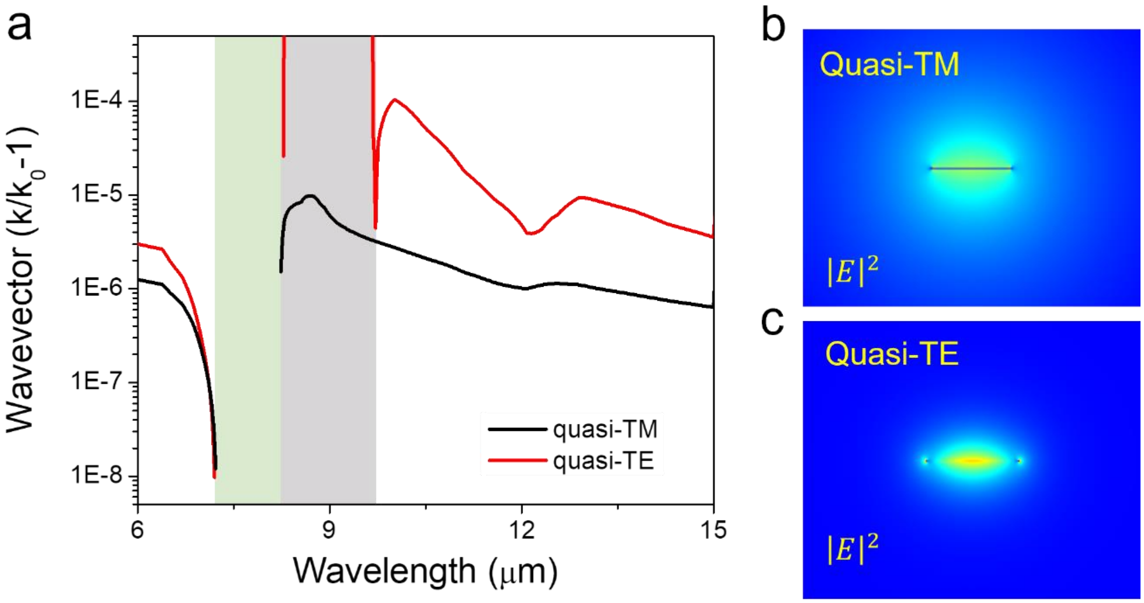

Fig. S8. **Wave vector and mode profile of quasi-TM and quasi-TE modes. a,** Numerical calculated wave vector of quasi-TM and quasi-TE modes of SiO_2 NR with 50 nm in thickness and 10 μm in width. The gray area marks the metallic regime of SiO_2 and the light green area

marks the region with $n + ik < 1$ where no mode is supported. **b, c,** The electric field distribution ($|\mathbf{E}|^2$) of each mode at $\lambda = 10\ \mu m$.

In order to gain a better understanding of how the waves propagate from the SiO_2 NR to the sensing beam and gradually get absorbed, we also conducted mode analysis on the sensing beam as shown in Fig. S9. The result shows that as the Fe_3O_4 thickness increases to over 300 nm, the propagation length of the waves greatly reduces to < 50 μm. In the experiment, we used 350 nm thick Fe_3O_4, which resulted in a short SPhP propagation length (Fig. 1e in the main text) and consequently enhanced the absorption coefficient of the SPhP by the reservoirs (the η_n term in Eq. S11). Of course, the final efficiency η_n is also depended on how much energy is coupled to the sensing beam, i.e., coupling efficiency. The analysis on the coupling efficiency is shown in Supplementary Note 9.

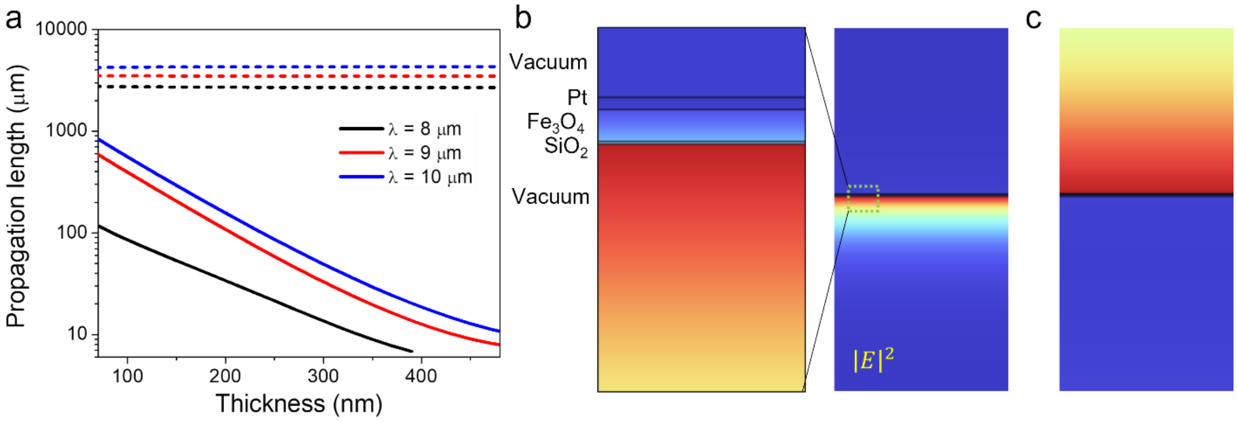

Fig. S9. **Propagation wave on the sensing beam. a,** Propagation length of two supported modes (solid and dash curve) as a function of Fe_3O_4 thickness. The corresponding electric field ($|\mathbf{E}|^2$) distributions are shown in **b** (solid) and **c** (dash). The thickness of Pt, Fe_3O_4 and SiO_2 are 110 nm, 350 nm, and 21 nm respectively.

Supplementary Note 8: Upper bound value of SPhP contributed thermal conductance

The equation derived from the previous analysis reveals that G_{SPhP} of the SiO_2 NR is dependent on the transmission probability τ_n and corresponding absorption coefficient η_n of each SPhP mode. We calculated the propagation length (L_{SPhP}) of quasi-TM and quasi-TE mode through numerical mode analysis, from which we can obtain the transmission probability as:

$$\tau_n(\omega) = \exp\left(-\frac{L}{L_{SPhP,n}(\omega)}\right) \quad \text{(S13)}$$

The absorption efficiency η_n, however, highly depends on the specific geometry and material properties. Computational complexity makes it difficult to be estimated accurately, especially considering that the feature size is only a few tens of nanometers while the whole device size is over a hundred micrometers. By assuming 100% absorption in the thermal reservoirs ($\eta_n = 1$), we can calculate the upper bound value of SPhP contributed thermal conductance G_{SPhP}. The thermal conductance and conductivity with considering the contributions of these two SPhP modes (quasi-TM and quasi-TE) are shown in Fig. S10. It can be seen that for a narrow ribbon of $W = 2$ µm, G_{SPhP} is linearly dependent on the temperature because of the one-dimensional transport behaviour when $W < \lambda$. The two curves in Fig. S10a overlap because the propagation lengths of the SPhP modes are long so that the SPhP contributions make no difference for nanoribbons with lengths of 50 µm and 100 µm. The corresponding conductivity values contributed by SPhP modes are shown in Fig. S10b.

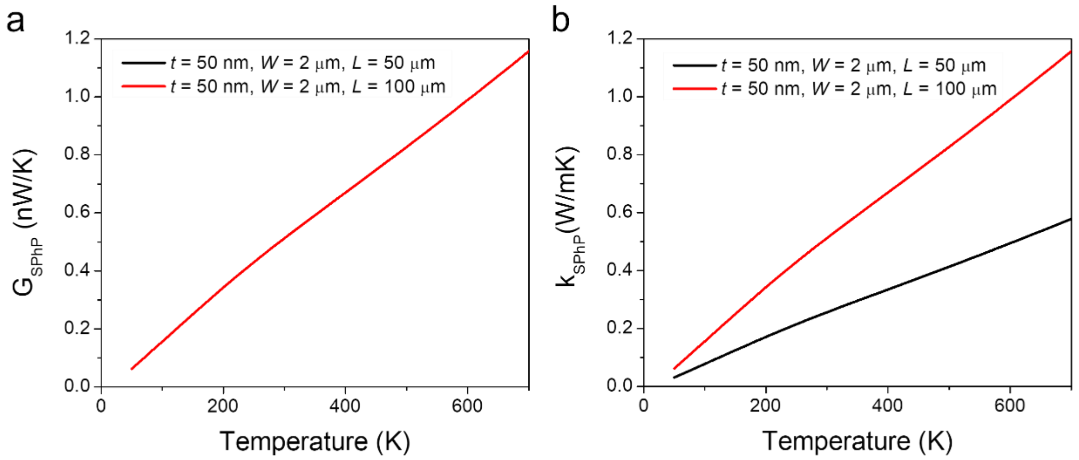

Fig. S10. SPhP contributed thermal conductance and conductivity. a, SPhP contributed thermal conductance for nanoribbons of t = 50 nm, W = 2 µm, L = 50 µm (black) and t = 50 nm, W = 2 µm, L = 50 µm (red). The black curve overlaps with the red curve. **b,** The corresponding thermal conductivity contributed by SPhP.

Supplementary Note 9: Mode size for absorption efficiency

The mode size can have significant implications for various aspects of waveguide performance, such as coupling efficiency, propagation loss, and susceptibility to environmental influences. Unlike cylindrical or square waveguides, the mode distribution of the SiO_2 NR is not isotropic. As shown in Fig. S11, the decay of the field intensity is faster in the vertical direction of the NR compared to the horizontal direction for a NR with $W > t$. One advantage of this design is that even though the overall mode volume is large, the mode size in the vertical direction can still be relatively small, thereby reducing the leakage to the substrate and improving the coupling efficiency to the sensing beam (reservoir). Here we define the mode size d as the distance from the interface where the field intensity is reduced to $1/e$ of the value at the solid surface (Fig. 3b in the main text). The calculated mode sizes in the vertical direction of SiO_2 NR are shown in Fig. S11.

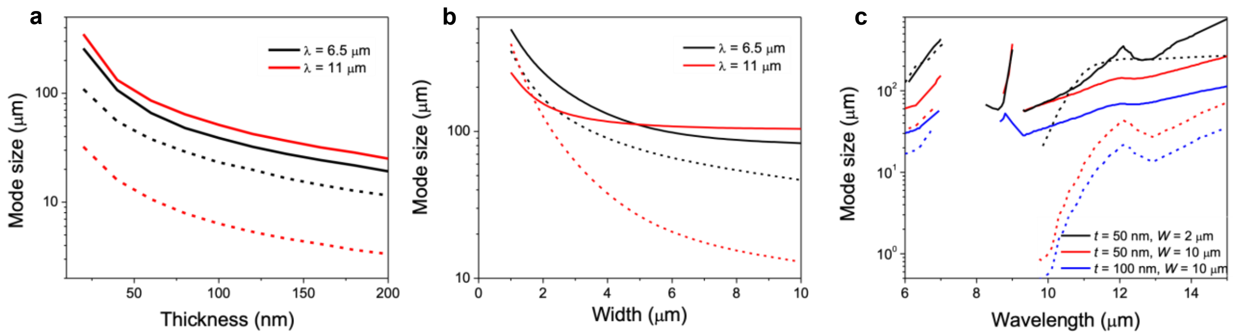

Fig. S11. SPhP mode volume of SiO_2 NR with different dimensions. a, SPhP mode size of NR with 10 μm width and different thickness. **b,** SPhP mode size of NR with 50 nm thickness and different width. **c,** Dependence of SPhP mode size on wavelength (solid curve: quasi-TE mode, dash curve: quasi-TM mode). Inset: SEM image showing the gap between suspended SiO_2 NR and Si substrate.

The overall absorption efficiency depends on the leakage during propagation, coupling efficiency from the SiO_2 NR to the sensing beam, and how much of this energy is eventually absorbed as heat. The SiO_2 NRs support two propagation modes as quasi-TM mode and quasi-TE mode. The sensing beam also supports two modes, one with a long propagation length and a large mode size, the other with a short propagation length and a small mode size (see Fig. S9). Each of the two modes on the SiO_2 NR can be coupled to either one of the two modes on the sensing beam, i.e., four possible coupling pathways, as shown as A, B, C and D in Fig. S12. The total coupling efficiency from quasi-TM mode and quasi-TE mode are $\eta_{quasi-TM} = \eta_A + \eta_B$ and $\eta_{quasi-TE} = \eta_C + \eta_D$. The terms η_A and η_C correspond to the couplings to the short propagation mode on sensing beam and end up with high absorption and high coupling efficiency when the mode size on SiO_2 NR is small. The terms η_B and η_D correspond to the couplings to the long propagation mode on the sensing beam and contribute less to the thermal conductance due to low absorption efficiency. We can neglect

the contributions from η_B and η_D and simply use η_A and η_C to represent the contributions from quasi-TM and quasi-TE modes to the thermal conductions (marked as the red arrows in the Fig. S12), i.e., $\eta_{quasi-TM} \approx \eta_A$ and $\eta_{quasi-TE} \approx \eta_C$. Please note that, from the coupling efficiency point of view, these four possible couplings have different coupling efficiencies depending on the mode similarity coefficient. For example, the SiO_2 NR with a large cross section has a smaller mode size and is more likely to couple to the mode on sensing beam with a small mode size, i.e., the couplings corresponding to the red arrows in Fig. S12. Considering that after we added a lossy Fe_3O_4 layer on the heating and sensing beams to enhance the absorption efficiency, the propagation length on the sensing beam reduced to a few micrometers to tens of micrometers, we assume that all the energy coupled to the short propagation mode on sensing beam is eventually absorbed. The coupling efficiency can be calculated with the mode profile by using coupled mode theory as[6,7]:

$$\eta = \frac{\omega\epsilon_0 \int_{-\infty}^{\infty}\int_{-\infty}^{\infty}(n_1^2 - n_2^2)\mathbf{E}_1^* \cdot \mathbf{E}_2 dxdy}{\int_{-\infty}^{\infty}\int_{-\infty}^{\infty} n_z \cdot (\mathbf{E}_1^* \times \mathbf{H}_1 + \mathbf{E}_1 \times \mathbf{H}_1^*)dxdy} \tag{S14}$$

where $\mathbf{E}_1(x,y)$, $\mathbf{E}_2(x,y)$, and $\mathbf{H}_1(x,y)$ are electric and magnetic fields in modes of waveguide 1 (SiO_2 NR) and waveguide 2 (sensing beam). $n_1(x,y)$ express the cross-sectional refractive index distribution in the case that both waveguides exist (SiO_2 NR and sensing beam), $n_2(x,y)$ is for the case that only the second waveguide (sensing beam) exists. ω, ϵ_0, and n_z are the angular frequency of the light wave, the permittivity of vacuum, and the unit vector along the propagation direction z.

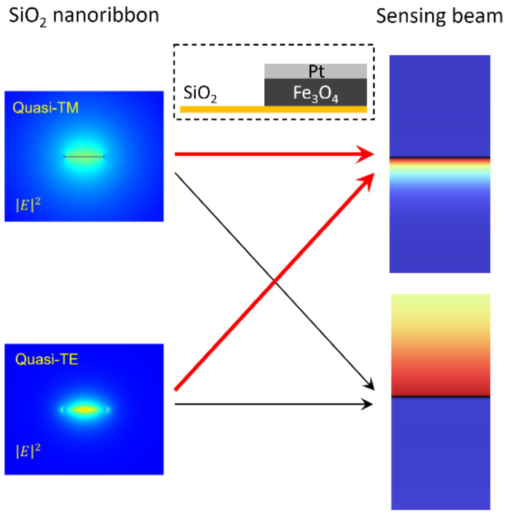

Fig. S12. **Possible mode couplings from the SiO_2 NR to the sensing beam.** Each of the two modes on the SiO_2 nanoribbons can be coupled to either one of the two modes on the sensing beam, i.e., four possible coupling pathways which are marked as A, B, C and D, respectively. The red line arrows mark the two couplings that make the dominant contribution

to the thermal conductance. The inset in the dash line box shows the cross section of our structure.

To obtain a qualitative dependence, we can use a semi-analytic method, namely, the effective index method, to get the mode profile on SiO_2 NR. By this method, a rectangular waveguide problem is transformed into a 1D effective waveguide problem. For our case of SiO_2 NR with thickness t and width W, we first solve the effective index n_{eff} of a SiO_2 slab with thickness of t, then use this n_{eff} for the second slab waveguide with width W to get the mode index. The solution for a general slab waveguide gives the mode size (MS) as[7,8]:

$$MS(\omega) \propto \frac{1}{\tanh\left(\frac{1}{2}k_0 t\sqrt{\epsilon_m(\omega) - \epsilon_d(\omega)}\right)}$$

(S15)

where ϵ_m and ϵ_d are the permittivity of the waveguide core and surrounding medium. For our case of SiO_2 NR, we can have the mode size as:

$$MS_{NR}(\omega) \propto \frac{1}{\tanh(tW/C(\omega))}$$

(S16)

where C is a parameter related to frequency and material property. The hyperbolic tangent function (tanh) means that the mode size approach to the unity when the cross-section tW increase, assuming that the mode profiles on SiO_2 NR and the mode profile on sensing beam are perfectly matched at this condition. Combining equation S14 and equation S16 with $E(x, y) = E_0 e^{-\frac{y}{2MS_{NR}}}$, and simplifying, we can express the coupling efficiency as:

$$\eta_n(\omega) \approx \tanh\,(tW/C(\omega))$$

(S17)

The parameter C originated from the optical material property, which is frequency dependent. However, the spectral dependence of the mode size is weak outside the Reststrahlen band. For SiO_2, the material property could vary dramatically within the Reststrahlen band but changes slowly with wavelength outside the Reststrahlen band, so does the mode size as shown in Fig. S11. The modes on the SiO_2 NRs are supported with a frequency range much broader than the Reststrahlen band, so the average mode size does not change strongly with the wavelength, especially at a high temperature. For example, at the temperature of 550 K, the corresponding thermal emission peak is about 5.27 μm which is already away from the Reststrahlen band.

We used $\eta_n = \tanh\,(A/C_{eff})$ to fit the data for our 1D samples (red data points in Fig.3d), where $A = tW$ is the cross section and C_{eff} is an effective parameter after considering the

frequency dependence. The trend aligns well when a single value of $C_{eff} = 0.45\ \mu\mathrm{m}^2$ is used (Fig. 3d in the main text). The fitted value of C_{eff} for our experiment data can be treated as an average scaling factor of the mode size for the four samples under the temperature of 550K.

Supplementary Note 10: Fe_3O_4 absorber

The Fe_3O_4 absorber in our sample was deposited by RF sputtering using a Fe_3O_4 target. From the SEM image (Fig. S13), its surface is porous and sparse. The color of the film on clear glass slides is dark red instated of the black color of Fe_3O_4. We measured the electric resistance of the Fe_3O_4 film using a 4-point probe method. The deposited Fe_3O_4 film electric resistance is around 24.4 $\Omega.\mathrm{cm}$, which is larger than pure Fe_3O_4 (~$5 \times 10^{-4}\ \Omega.\mathrm{cm}$) [9] and smaller than pure Fe_2O_3 (~$3 \times 10^{4}\ \Omega.\mathrm{cm}$) [10]. We can assume that the composition of our film is a mixture of both, i.e., $(FeO)_x\ (Fe_2O_3)_{1-x}$. This is likely caused by the residual oxygen in the sputter vacuum chamber.

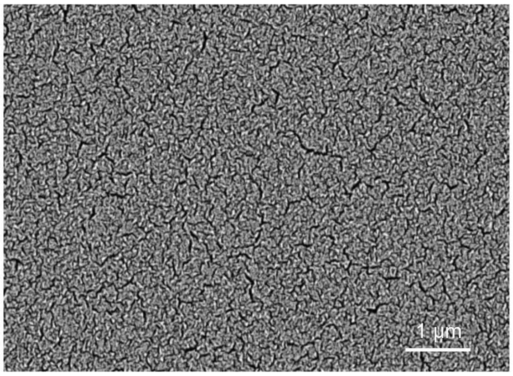

Fig. S13. SEM image of sputter deposited Fe_3O_4 film.

Supplementary Note 11: SiO_2 NR emissivity

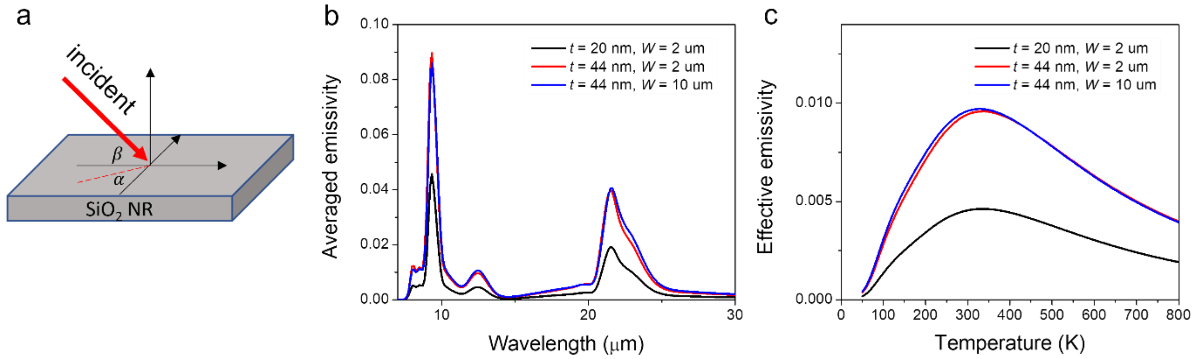

Fig. S14. Simulation result for the emissivity of the thin SiO_2 NRs. a, Schematic of the simulated structure calculating the absorption of incident light from varies directions by the SiO_2 NR. **b,** Directionally averaged emissivity vs. wavelength for NRs with different thicknesses and widths. **c,** Total effective emissivity vs. temperature.

To obtain the surface emissivity of the SiO_2 NR, we performed full-wave simulations by using the commercial software COMSOL Multiphysics. The SiO_2 NR was set to be infinitely long using periodic boundary conditions at two ends and surrounded by vacuum with a perfect matching layer (PML) as the boundary condition to ensure accuracy. The incident light was modeled as a plane wave with a certain angle of incidence (α, θ) (Fig. S14a). The absorption cross-section was calculated as:

$$\sigma_{abs}(\alpha, \theta, \lambda) = \frac{Q_{abs}(\alpha,\theta,\lambda)}{I_0} \quad \text{(S18)}$$

Where Q is the total absorption obtained by integrating the Ohmic losses over the volume of SiO_2 NR, I_0 is the intensity of incident light, (α, θ) stands for the angle of incident, λ is the wavelength of incident light. Then, by using the Kirchhoff's law, the emissivity can be expressed as:

$$\epsilon(\alpha, \theta, \lambda) = \frac{\sigma_{abs}(\alpha,\theta,\lambda)}{S} \quad \text{(S19)}$$

where S is the physical surface area. In simulations, we performed 200-300 different incident angles for each structure to obtain the averaged emissivity $\overline{\epsilon}(\lambda)$ (Fig. S14b). The temperature dependent effective emissivity (Fig. S13c) can be calculated as:

$$\epsilon(T) = \frac{\int \mathrm{BB}(T,\lambda)\cdot\overline{\epsilon}(\lambda)d\lambda}{\int \mathrm{BB}(T,\lambda)d\lambda} \quad \text{(S20)}$$

where $\mathrm{BB}(T, \lambda)$ is the black-body radiation based on Planck's law.

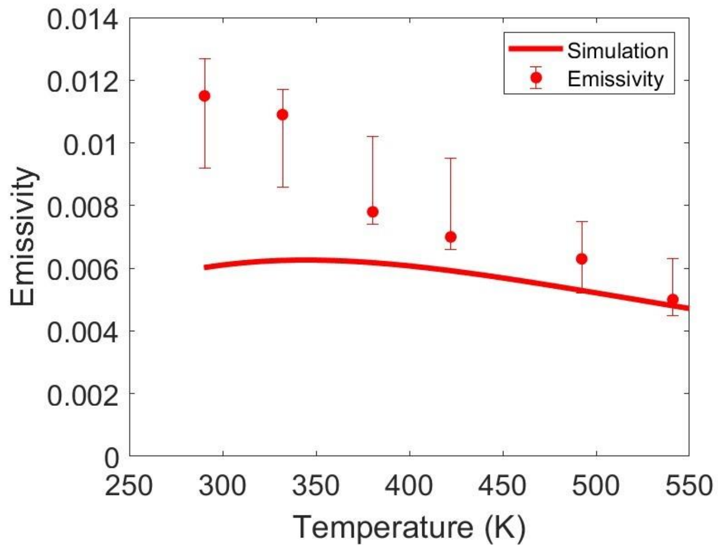

Fig. S15. Comparison between the simulated results and the experimentally extracted emissivity from the 400 μm long NR sample (shown in Fig. 2d in the main text). The NR thickness is 30 nm. The error bars for emissivity are derived from the errors of the thermal conductivity in Fig. 2d.

Fig. S15. shows a comparison between the simulated results and the experimentally extracted emissivity from the 400 μm long NR sample in Fig. 2d. The NR thickness is 30 nm in this case. The simulation result agrees well with the extracted value at high temperatures (> 350 K). At lower temperature, the simulation result underestimates the emissivity value. This is likely due to the more leakage of the SPhP waves to the substrate at lower temperatures. At lower temperatures, the peak thermal wavelength is longer and therefore the corresponding mode sizes of the propagating modes are generally larger, leading to more leakage. This part of the energy loss is included in the experiments but is not accounted for in the simulation, since it is very challenging to handle such a large simulation domain.

Supplementary Note 12: Radiation heat loss from NR

During the measurement, the whole chamber environment including the Si substrate is carefully controlled at the same environmental temperature T, which varied from 300 K to about 550 K in this study. Therefore, we can consider the ribbon is being surrounded by an enclosure at the same temperature (T). Since the ribbon is much smaller than the surrounding objects, include the vacuum chamber wall and the Si substrate, we can assume the surrounding enclosure as a blackbody, regardless of the actual optical properties or emissivity of the surrounding materials. Radiative heat transfer flux between two gray surfaces is[11]:

$$\dot{Q}_{12} = \frac{\sigma A_1\left(T_1^4 - T_2^4\right)}{\frac{1-\varepsilon_1}{\varepsilon_1} + \frac{1}{F_{12}} + \frac{A_1}{A_2}\frac{(1-\varepsilon_2)}{\varepsilon_2}} \tag{S21}$$

where A and ε are the surface area and emissivity respectively, σ is the Stefan–Boltzmann constant. Subscripts 1 and 2 means the objects 1 and 2. In our case of a very small nanoribbon (object 1) surrounded by a much larger enclosure (object 2), $F_{12} = 1,$ and $\frac{A_1}{A_2} \cong 0$. The emissivity of nanoribbon ε_1 is very small. Therefore, the above equation is reduced to:

$$\dot{Q}_{12} = \varepsilon_1 A_1 \sigma(T_1^4 - T_2^4) \tag{S22}$$

We can see that the radiative flux only depends on the emissivity of object 1 (i.e., nanoribbon in our case) and is independent of the properties of the enclosure (either the vacuum chamber wall or the Si substrate).

As mentioned earlier, $T_2 = T$ (enclosure or environmental temperature), $T_1 = T + \Delta T$ where $\Delta T \ll T$ (Note: ΔT is between ΔT_h and ΔT_s), so the radiative heat transfer coefficient (h_r) can be written as:

$$h_{\mathrm{r}} = \frac{\varepsilon_1 A_1 \sigma\left(T_1^4 - T_2^4\right)}{T_1 - T_2} \cong 4\varepsilon_1 \sigma T^3 \tag{S23}$$

Where ε_1 is the NR emissivity, which is obtained from the simulation described in Note 11 for different NR thicknesses. The radiation heat loss from the NR is calculated based on a thermal fin model. The steady state heat conduction equation along the NR can be written as:

$$kA\frac{d^2\Delta T}{dx^2} - h_{\mathrm{r}} P \Delta T = 0 \tag{S24}$$

where *k* is the NR thermal conductivity, *A* is the cross-section area of the NR and *P* is the perimeter of the NR cross section.

By setting $\beta^2 = \frac{hP}{Ak}$, the Eq. S24 can be written as:

$$\frac{d^2\Delta T}{dx^2} = \beta^2 \Delta T \tag{S25}$$

The general solution for Eq. S23 is

$$\Delta T = C_1 \sinh(\beta x) + C_2 \cosh(\beta x) \tag{S26}$$

After applying the boundary condition

$$\Delta T|_{x=0} = \Delta T_{\mathrm{h}} \tag{S27}$$

$$\Delta T|_{x=L} = \Delta T_{\mathrm{s}} \tag{S28}$$

where L is the NR length, we can get the temperature distribution along the NR as follows:

$$\Delta T(x) = \left(\Delta T_s - \frac{\Delta T_{\mathrm{h}} \cosh(\beta L)}{\sinh(\beta L)} L\right) \sinh(\beta x) + \Delta T_{\mathrm{h}} \cosh(\beta x) \tag{S29}$$

The total radiation heat loss from the NR can be calculated by integrating the heat loss along the NR:

$$Q_{loss} = \int_0^L hP\Delta T(x)dx \tag{S30}$$

Supplementary Note 13: Device background thermal conductance

In order to account for the device background conductance (G_{bg}) that comes from direct radiation heat transfer between the heating and sensing beams, we measured the conductance on devices without NR between the two beams. As can be seen from Fig. S16a, this background thermal conductance increases rapidly with temperature, and it is not neglectable at high temperature. This background thermal conductance is much larger than the beam-to-beam blackbody radiation thermal conductance (G_{BB}) as shown in the Fig. S16a, where G_{BB} is calculated using:

$$G_{BB} = 4\varepsilon\sigma A_{side}F_{12}\left(\frac{T_h+T_s}{2}\right)^3 \quad \text{(S31)}$$

where $\varepsilon = 1$ was used to calculate the blackbody limit, A_{side} is the side wall surface area of the beam facing each other (which is the product of the thickness and the length of each beam), F_{12} is the view factor for the two parallel side walls of the beams (for the 44 μm gap, F_{12} = 0.0045, and for the 94 μm gap F_{12} = 0.0018). T_h and T_s are the temperature of the heating and sensing beam, respectively. The measured background conductance much higher over the blackbody limit is due to the directional thermal radiation mediated by SPhP in the thin SiO_2 beams, as studied by Reddy et al [12].

We also found that the background conductance decreased rapidly as the gap size increased. For the 400 μm gap, there was no measurable background signal at all. Instead of the linear relation between ΔT_s and heating power, we could only see noises in the ΔT_s measurement, as shown in Fig. S16b.

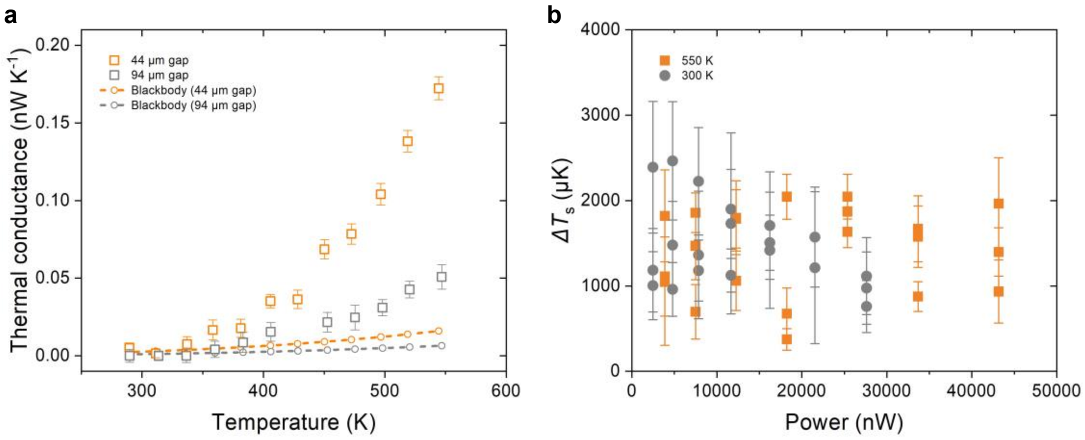

Fig. S16. **The background conductance for devices of different gaps. a,** Background thermal conductance for devices without a NR. **b,** ΔT_s as a function of heating power for 400 μm gap device, showing only noise with this gap size, meaning no measurable background conductance.

In addition, the background signal can be removed directly during the measurement as shown in Supplementary Note 16.

Supplementary Note 14: Extracting the SPhP conductance

In order to determine the contribution of the propagating SPhP to thermal conductance, it is necessary to eliminate the effects of other factors, such as the phonon thermal conductance of SiO_2 NR, background conductance, and radiation heat loss, from the measured apparent thermal conductance. First, the temperature distribution on the NR was obtained using the thermal fin model, and the radiation heat loss (Q_{loss}) was calculated by integrating the heat dissipation over the entire length of the NR (Eq. S30 in Supplementary Note 12). Next, we calculated the heat flux conducted by phonon (Q_{ph}) using the NR geometry and the bulk phonon thermal conductivity value of SiO_2 [13]. The effective thermal conductance of phonon with the radiation heat loss was then calculated as:

$$G_{ph+loss} = \frac{Q_{ph} - Q_{loss}}{\Delta T_h - \Delta T_s} \quad \text{(S32)}$$

where ΔT_h and ΔT_s are the experimentally measured temperature rises of the heating and sensing sides respectively. Additionally, the background thermal conductance (G_{bg}) was determined by measuring the thermal conductance of a blank device without SiO_2 NR between the heating and sensing beam (Supplementary Note 13 and Fig. S16). Consequently, the SPhP thermal conductance is extracted as:

$$G_{SPhP} = G_{Apparent} - G_{ph+loss} - G_{bg} \quad \text{(S33)}$$

Supplementary Note 15: Thickness and width-dependence of SPhP thermal conductance

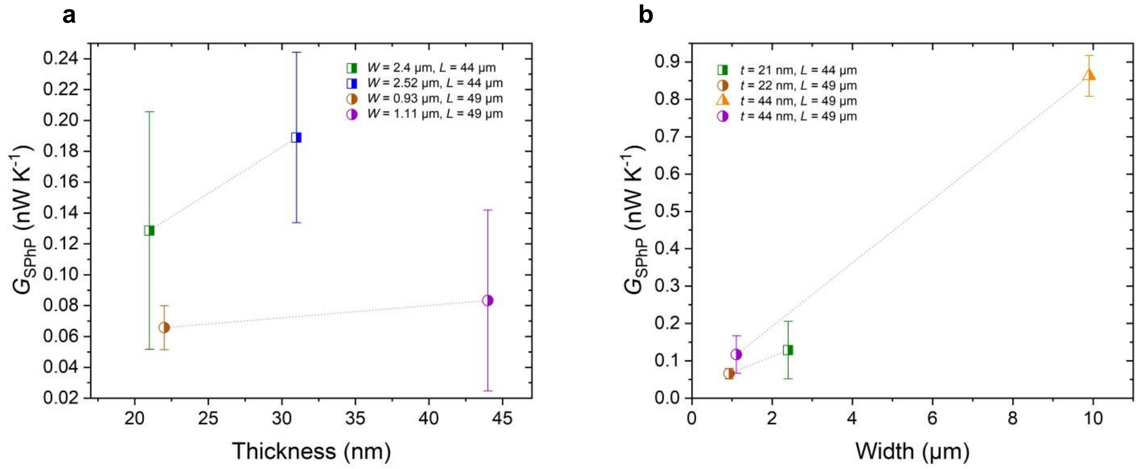

Fig. S17. Thickness and width-dependence of SPhP thermal conductance. a, Thickness, and **b,** width-dependence of SPhP thermal conductance of SiO_2 NRs.

Supplementary Note 16: Background canceling measurement

The device background single as mentioned in Supplementary Note 13 can be directly eliminated though the ‘canceling’ bridge circuit as shown in Fig. S18. A blank pair device without a nanoribbon and a pair resistance $R_{h,2}$ have been added to the original measurement platform. An identical heating current (I_h) is applied to the heating sides (R_h and $R_{h,p}$) of both devices to create the same temperature rise in the two heating beams. Since the devices are almost identical and their background conductance values are approximately equal. The difference in the resistance changes on R_s and $R_{s,p}$ which corresponding to the temperature rises on the sensing sides of the two devices is directly measured using a Wheatstone bridge. Consequently, we can directly obtain the nanowire conductance from a single measurement based on this scheme.

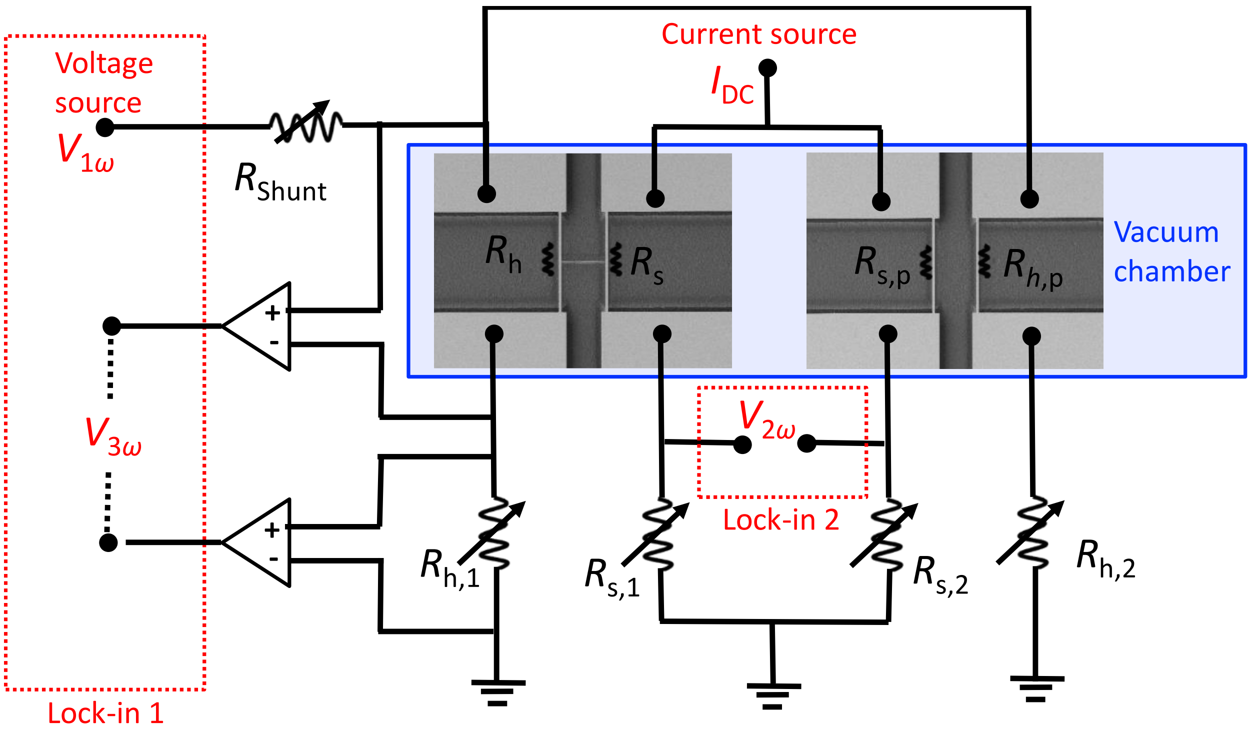

Fig. S18. Schematic of the background signal canceling measurement platform.

A SiO_2 NR (t = 54 nm, W = 9.98 µm, L = 50 µm) has been measured with (Fig. S18) and without (Fig. S4) background canceling circuit, separately. From Fig. S19a, it can be seen that the apparent thermal conductivity still shows enhancement at high temperatures after removing the background signal. For the apparent thermal conductance measured with background signal canceling, SPhP thermal conductance is calculated by

$$G_{SPhP} = G_{Apparent} - G_{ph+loss} \qquad \text{(S34)}$$

While for another measurement method, the SPhP thermal conductance is extracted as:

$$G_{SPhP} = G_{Apparent} - G_{ph+loss} - G_{bg} \qquad \text{(S35)}$$

Fig. S19b shows the comparison of G_{SPhP} of the same sample with different measurement methods. Either removing the background signal directly in the measurement or subtracting the background signal during data processing, the calculated G_{SPhP} were similar which validates our approach to removing background signal in Supplementary Note 14.

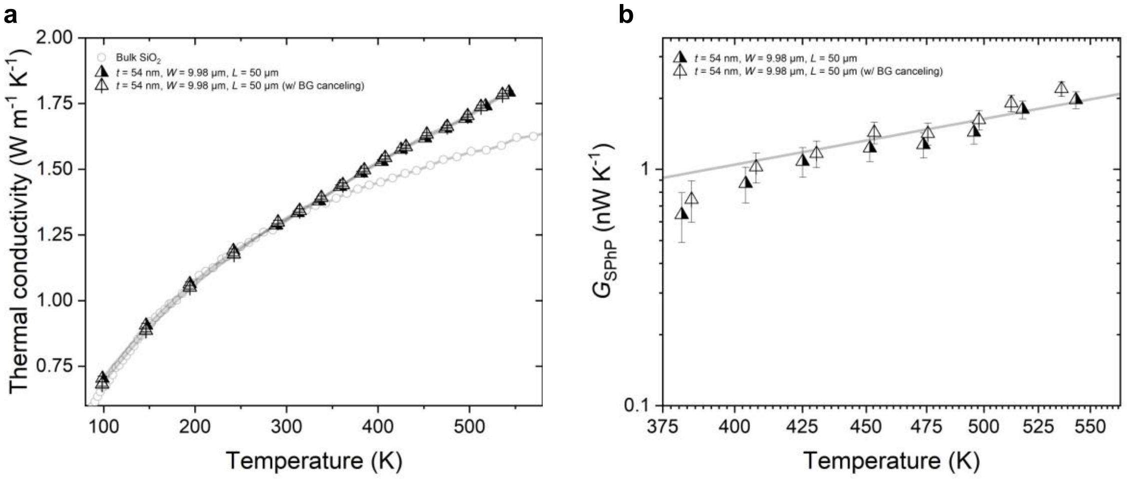

Fig. S19. Background signal canceling measurement results. a, The apparent thermal conductivity and **b,** the calculated SPhP thermal conductance of the SiO_2 NR measured with and without BG canceling circuit, separately.

References

1 Wingert, M. C., Chen, Z. C., Kwon, S., Xiang, J. & Chen, R. Ultra-sensitive thermal conductance measurement of one-dimensional nanostructures enhanced by differential bridge. *Review of Scientific Instruments* **83**, doi:Artn 024901 10.1063/1.3681255 (2012).

2 Shin, S. M., Elzouka, M., Prasher, R. & Chen, R. K. Far-field coherent thermal emission from polaritonic resonance in individual anisotropic nanoribbons. *Nature Communications* **10**, doi:ARTN 1377 10.1038/s41467-019-09378-5 (2019).

3 Landauer, R. Spatial Variation of Currents and Fields Due to Localized Scatterers in Metallic Conduction. *Ibm J Res Dev* **1**, 223-231, doi:DOI 10.1147/rd.13.0223 (1957).

4 Tranchant, L. *et al.* Two-dimensional phonon polariton heat transport. *Nano letters* **19**, 6924-6930 (2019).

5 Gluchko, S., Palpant, B., Volz, S., Braive, R. & Antoni, T. Thermal excitation of broadband and long-range surface waves on SiO2 submicron films. *Applied Physics Letters* **110** (2017).

6 Yariv, A. Coupled-mode theory for guided-wave optics. *IEEE Journal of Quantum Electronics* **9**, 919-933 (1973).

7 Okamoto, K. *Fundamentals of optical waveguides*. (Elsevier, 2021).

8 Knox, R. & Toulios, P. in *Proc. Symp. Submillimeter Waves.* 497-515 (Brooklyn, NY).

9 Martinez, A. I., Garcia-Lobato, M. A. & Perry, D. L. Study of the Properties of Iron Oxide Nanostructures. *Nanotechnol Sci Tech*, 183-194 (2009).

10 Saritaş, S., Turgut, E., Kundakci, M., Gürbulak, B. & Yildirim, M. Compared with a-Fe2O3 and ZnXFe3-XO4 thin films grown by Chemical Spray Pyrolysis. *Int J Sens Netw Data Commun* **6**, 2 (2017).

11 Bergman, T. L., Lavine, A. S., Incropera, F. P. & DeWitt, D. P. *Introduction to heat transfer*. (John Wiley & Sons, 2011).

12 Thompson, D. *et al.* Hundred-fold enhancement in far-field radiative heat transfer over the blackbody limit. *Nature* **561**, 216-+, doi:10.1038/s41586-018-0480-9 (2018).

13 Cahill, D. G. Thermal-Conductivity Measurement from 30-K to 750-K - the 3-Omega Method. *Review of Scientific Instruments* **61**, 802-808, doi:10.1063/1.1141498 (1990).
